# Evidence for Crystalline Structure in Dynamically-Compressed Polyethylene up to 200 GPa

**DOI:** 10.1038/s41598-019-40782-5

**Published:** 2019-03-12

**Authors:** N. J. Hartley, S. Brown, T. E. Cowan, E. Cunningham, T. Döppner, R. W. Falcone, L. B. Fletcher, S. Frydrych, E. Galtier, E. J. Gamboa, A. Laso Garcia, D. O. Gericke, S. H. Glenzer, E. Granados, P. A. Heimann, H. J. Lee, M. J. MacDonald, A. J. MacKinnon, E. E. McBride, I. Nam, P. Neumayer, A. Pak, A. Pelka, I. Prencipe, A. Ravasio, M. Rödel, K. Rohatsch, A. M. Saunders, M. Schölmerich, M. Schörner, A. K. Schuster, P. Sun, T. van Driel, J. Vorberger, D. Kraus

**Affiliations:** 1 Helmholtz-Zentrum Dresden-Rossendorf, 01328 Dresden, Germany; 2 Open and Transdisciplinary Research Institute, Osaka University, Suita, Osaka 565-0871 Japan; 3 SLAC National Accelerator Laboratory, Menlo Park, CA 94309 USA; 4 Technische Universität Dresden, 01062 Dresden, Germany; 5 Lawrence Livermore National Laboratory, Livermore, CA 94550 USA; 6 Department of Physics, University of California, Berkeley, CA 94720 USA; 7 Technische Universität Darmstadt, 64289 Darmstadt, Germany; 8 Centre for Fusion, Space and Astrophysics, Department of Physics, University of Warwick, Coventry, CV4 7AL United Kingdom; 9 European XFEL GmbH, 22869 Schenefeld, Germany; 10 GSI Helmholtzzentrum für Schwerionenforschung GmbH, 64291 Darmstadt, Germany; 11 LULI, CNRS-CEA, Universite Paris VI-Ecole Polytechnique, 91128 Palaiseau Cedex, France; 12 Institut für Physik, Universität Rostock, 18051 Rostock, Germany

## Abstract

We investigated the high-pressure behavior of polyethylene (CH_2_) by probing dynamically-compressed samples with X-ray diffraction. At pressures up to 200 GPa, comparable to those present inside icy giant planets (Uranus, Neptune), shock-compressed polyethylene retains a polymer crystal structure, from which we infer the presence of significant covalent bonding. The *A*2*/m* structure which we observe has previously been seen at significantly lower pressures, and the equation of state measured agrees with our findings. This result appears to contrast with recent data from shock-compressed polystyrene (CH) at higher temperatures, which demonstrated demixing and recrystallization into a diamond lattice, implying the breaking of the original chemical bonds. As such chemical processes have significant implications for the structure and energy transfer within ice giants, our results highlight the need for a deeper understanding of the chemistry of high pressure hydrocarbons, and the importance of better constraining planetary temperature profiles.

## Introduction

Carbon and hydrogen are among the most abundant elements throughout the universe and are major constituents of large planets^1–3^. The bodies in which carbon is found in significant quantities are known as the ‘ice giants’, as opposed to the gas giants - which are dominated by hydrogen and helium^4^ - or rocky planets, with minimal light element atmospheres^5^. In the mantles of the ice giants, mixtures of ammonia, water and hydrocarbons are found in complex warm dense states^6,7^: methane, a major constituent of such planets^8^, dissociates and polymerises at high pressures^9^, with the liberated hydrogen becoming metallic^10^, while water and ammonia form superionic states^7,11^. Because these conductive states give rise to the planetary dynamo, their structure might explain the unusual magnetic fields of Uranus and Neptune^12,13^. Understanding the properties of high pressure polymers is therefore directly relevant to the behaviour of planetary interiors within our own solar system, and throughout the galaxy^14^.

On Earth, the extreme conditions found in planetary deep interiors can only be reached transiently, by shock compression^15^ or, for lower pressure states, static compression and heating^16,17^. The samples for these experiments can be light, fluid hydrocarbons such as methane or ethanol, but to avoid the complications of containing such samples, plastics are generally preferred. As well as being used as surrogates for planetary interiors, plastics are also commonly used as ablator materials for laser-driven shock compression experiments^18^, and in inertial confinement fusion targets^19^.

Recent work at the Linac Coherent Light Source (LCLS)^20,21^ observed the formation of diamond in shock-compressed polystyrene (CH) at 150 GPa and 5000 K, validating the theoretical prediction of ‘diamonds in the sky’ within ice giants^22^. These results are evidence of carbon-hydrogen demixing and subsequent crystallization; such processes are only possible if the chemical bonds within the original CH have been broken due to the shock-induced temperature rise. Here, we show data for polyethylene (CH_2_) driven to similar pressure conditions, with the diffraction signal showing no evidence of diamond formation. Instead, the lower shock temperature allows a polymer structure to remain, resolving a disagreement between first-principles simulations^9,23^.

## Experiment

**Figure 1:**
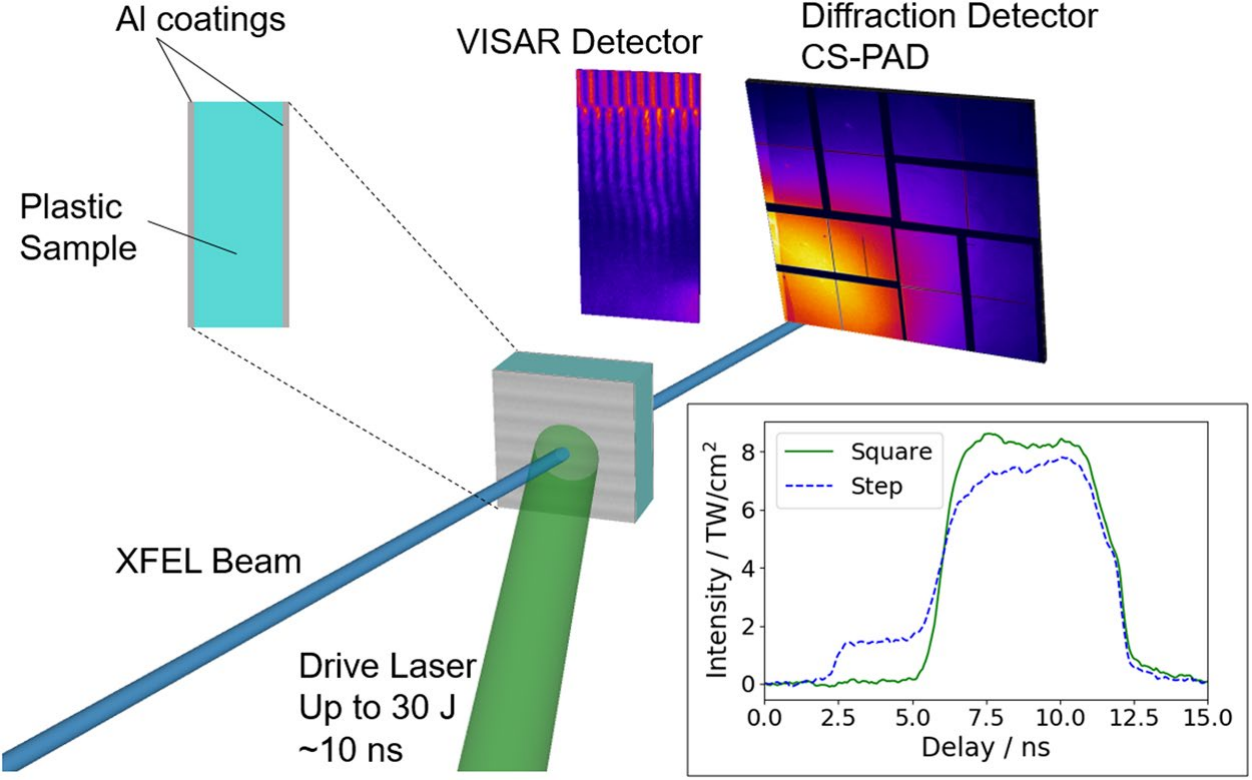
Schematic of the experimental setup at the Matter at Extreme Conditions endstation of LCLS. The high-energy laser beam irradiates the plastic sample, driving a shock wave into it. The conditions reached were monitored by a VISAR diagnostic, and the compressed sample was probed by a single X-ray pulse at either 8.1 or 8.2 keV. The scattered X-ray signal was observed by the large area CS-PAD detector. The inset shows example pulse shapes for square and step pulses, each averaged over four shots.

A schematic of the experimental setup is shown in Fig. 1. The drive laser, containing up to 30 J of energy, drove shocks into the CH foil. The sample was coated with aluminium (~100 nm) on each side, preventing sample disturbance from low intensity at early times and providing a reflective rear surface for the VISAR (Velocity Interferometer System for Any Reflector) probe laser^24^. As shown in the inset, the pulse shape was either square or stepped, in order to drive single or double shocks, respectively; the latter allowed high pressure conditions to be reached but with a much smaller rise in temperature than from a single shock with the same total pressure. At a given delay after the shock drive began, the sample was probed by the X-ray Free Electron Laser (XFEL) beam at an energy of 8.1 or 8.2 keV, and the diffraction from the sample observed on the Cornell-SLAC Pixel Array Detector (CS-PAD), covering an angular range of around 20°–90°. In general, the VISAR detector was not able to observe fringe shifts, as the sample was not reflective after shock breakout, but was able to monitor the breakout time and therefore the average shock speed; more details are given in the Methods sections of Ref.^20,25^.

**Figure 2:**
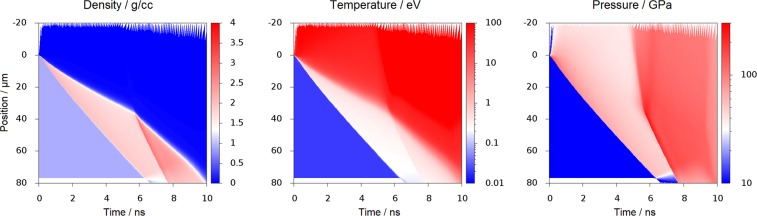
Simulated evolution of density, temperature and pressure for shock-compressed CH_2_ samples, driven by a laser incident from above. The driving laser parameters are taken from Ref.^20^, and had been optimised for polystyrene (CH) samples; consequently the two shocks do not reach the rear surface of the CH simultaneously. This is true for many of the shots reported in this work, although the intensity of the first shock was varied to bring them closer together in time. The simulations were performed using the radiation hydrodynamics code MULTI with SESAME equation of state 7171. The conditions in the double-shocked region are *ρ* = (2.4 ± 0.1) g/cc, *T* = (4,000 ± 400) K and *P* = (150 ± 15) GPa; these are similar to the conditions in polystyrene, apart from the significantly lower temperature.

The conditions in the sample were estimated from one-dimensional radiation hydrodynamics simulations, using the MULTI code^26^, using the SESAME equation of state (EOS) table 7171, and including radiation transport. The results of such a simulation are presented in Fig. 2; in this simulation, the laser drive had an initial intensity of 2.7 TW/cm^2^, rising to 7.1 TW/cm^2^ after 4.5 ns. This particular pulse shape had been calculated from a combination of the measured laser parameters and fitted to the observed sample response in CH, in order for the two shocks to break out of the sample simultaneously (see Methods of Ref.^20^). Due to the different EOS of CH_2_, the same laser driver does not give simultaneous breakout in this sample, and instead the sample has already begun to release pressure before the second shock reaches the rear surface.

In order to bring the breakout times in CH_2_ closer together, and to reach a wider variety of pressure-temperature states, the intensity of the first pulse was varied between 1.56 to 2.91 TW/cm^2^. Lower intensities were favoured as these gave a slower first shock, and therefore a smaller delay between the breakouts at the rear surface of the CH_2_. Since the majority of the temperature rise occurs during the first shock, changing the intensity of the first pulse significantly changes the temperature in the final state. Shots were also taken using only a single shock, both for calibration and to reach conditions along the shock Hugoniot. Although there was also significant random shot-to-shot variation in the drive laser energies, on the order of 10%, the energy on each individual shot was monitored, allowing shot-by-shot comparison of experiment and simulation. The primary sources of uncertainty in the conditions reached were the choice of EOS used and the effect of radiation in the simulation.

## Results and Discussion

**Figure 3:**
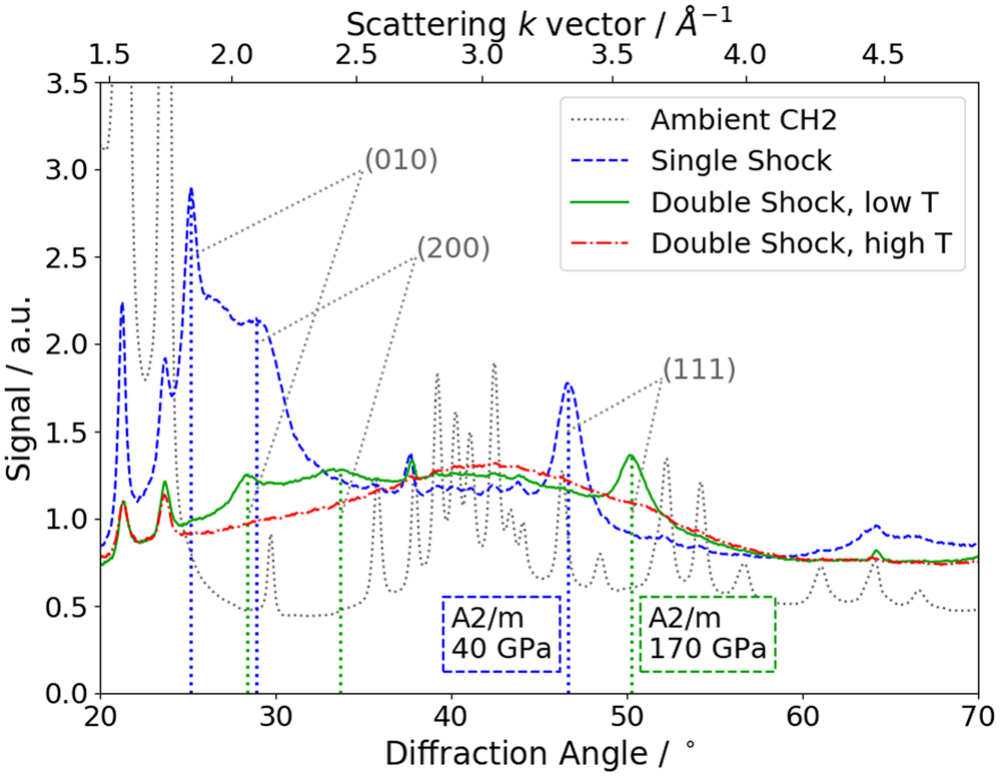
Diffraction data from CH_2_ samples at different conditions: preshock/ambient (grey dotted line), single shock (blue dashed line), double shock with weak first shock (green solid line) and double shock with strong first shock (red dot-dashed line). New peaks, due to the *A2/m* phase, are present for the single shock and first double-shocked lines; the positions of the first three diffraction lines at the best-fitting pressure are marked, and labeled with the relevant Miller indices.

The diffraction data was collected on the area detector and then azimuthally integrated for each shot to give an angle-resolved lineout, using the Dioptas software package^27^. The effects of XFEL polarization, as well as absorption in the targets and filters, were calculated and accounted for in the analysis. Examples of data from CH_2_ shots are shown in Fig. 3, as a function of diffraction angle *θ* and scattering k-vector *k* = (4*πλ*) sin (*θ*/2), with *λ* being the X-ray wavelength. The ambient data shows a complex crystal structure, primarily due to the *Pnam* space group crystal structure. Features of this phase, particularly the two strong peaks at 21.5° and 23.5°, are present in all of the laser-driven shots. This is due to a halo around the central X-ray spot, comprising around 5% of the total signal, which diffracts from ambient material. The signal from the shocked material is dominated by an amorphous liquid-like structure^25^, with no long-range order between the particles in the sample. However, many shots also clearly display new peaks, which were not present in the initial sample.

In the single shock case shown in Fig. 3, we can identify new peaks at scattering angles of 25°, 29° and 47°. Comparing these to diffraction signals seen in previous work on statically compressed CH_2_ samples^28–30^, they appear to correspond to the (010), (200) and (111) diffraction lines from a monoclinic *A*2*/m* structure. This structure had previously been reported up to pressures of 40 GPa in CH, and was estimated to be the most stable configuration for P > 33 GPa^30^.

For the shots taken with a double-shocked sample, examples are given in Fig. 3 with either a strong or weak initial shock, giving the lineouts labeled ‘Double Shock, high T’ and ‘low T’, respectively. The fomer is close to the conditions reached in the simulation of Fig. 2, as the second shock breaks out, while the latter was reached with a lower intensity drive for the first shock. In this lower temperature case, the (111) peak is again clearly visible above the amorphous background, while the weak peaks around 30° and 34° seen in the lineout only appear on some shots. In the higher temperature case, the sample is melted, such that no lattice remains, and only an amorphous liquid structure is observed.

**Figure 4:**
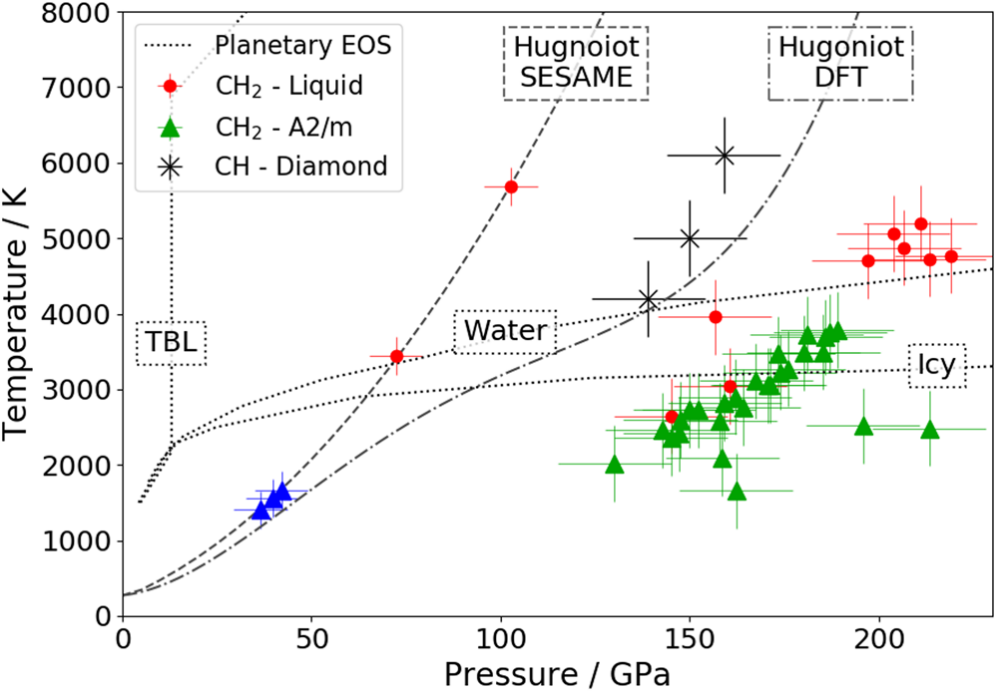
Pressure-temperature conditions reached, estimated from radiation hydrodynamics simulations with Sesame EOS 7171. Triangular points show where plastic structure remained after either a single shock (blue) or double shock (green), while the red points indicate shots where plastic structure disappeared. For comparison, the conditions at which diamond was observed to form from CH in^20^ are shown by black crosses. The Hugoniot lines indicate conditions reached by a single shock in CH_2_, calculated either by SESAME or DFT simulations^23^. Conditions estimated from different models of planetary interiors - thermal boundary layer (TBL)^37^, water-only^7^ and icy Uranus^35^ - are shown as dotted lines.

With the variety of conditions reached, we are able to observe the behaviour at a wide range of parameter combinations on the phase diagram, in order to see where plastic structure persists, as shown in Fig. 4. The triangles indicate shots where the *A*2*/m* structure was at least partially observed i.e. the (111) diffraction peak at around 50° was seen, with the colours corresponding to the single shock (blue) or low temperature double shock (green) cases in Fig. 3; similarly, the red points indicate conditions where no new Bragg peaks were seen. The presence of shots without crystalline peaks close to 150 GPa and 3000 K suggests that we are near the edge of the stability region of the *A2/m* structure. The melt line moves to higher temperatures with increasing pressure, although the uncertainties in our conditions mean that it cannot be characterised precisely.

The black crosses in Fig. 4 show the pressure-temperature conditions at which diamond formation from CH samples was observed, as previously reported^20^. For demixing to have occurred in CH, the bonds between the carbon and hydrogen must have been broken, or at least sufficiently weakened that order between carbon and hydrogen has been lost and the carbon atoms could rearrange into the diamond lattice. Due to the lower temperature, inferred from the radiation hydrodnamics simulations shown in Fig. 2, this bond breaking is not happening as quickly, or completely, in the CH_2_ sample. Instead, lattice structures of polymers, rather than from diamond, are seen.

The conditions reached here, as well as their associated uncertainties, are estimated using the SESAME EOS. However, other EOS models may suggest rather different conditions. At pressures along the shock Hugoniot, Mattsson *et al*.^23^ estimated the densities and temperatures in CH_2_ using density functional theory molecular dynamics (DFT-MD), finding much lower temperatures than given by SESAME, as shown by the grey Hugoniot lines in Fig. 4. This is in contrast to CH in which, at the pressures considered here, there is much better agreement between first-principle simulations, the SESAME EOS and experimental results^31,32^. The simulations of Mattsson also predict that, along the Hugoniot, C-H dissociation in CH_2_ becomes significant for pressures of between 70–100 GPa; our results show the disappearance of lattice, and therefore polymer, structure at similar pressures (72 ± 7 GPa), although we have only a limited number of single-shock shots. How this different EOS would affect the temperatures reached in our double-shocked experiments is not clear, as the conditions only remain on the Hugoniot to low pressures (up to 50 GPa), where the temperature difference is small. Since direct measurement of the temperature is very difficult in experiments, it was not attempted.

Turning to a more detailed analysis of the *A*2*/m* phase behaviour, we now consider only the shots where three diffraction lines are observed; these together allow us to determine the lattice parameters, while the single (111) peak is insufficient. We first note that, unlike what would be predicted for a monoclinic structure, the separation of the (111) and (−111) lines was never observed. This fact implies that the angle *β* in the structure is approximately 90°, such that the structure reduces to orthorhombic, rather than monoclinic. The lattice parameters measured by Fontana *et al*. at 44 GPa^30^, with *β* = 88°, would imply a separation of 0.5° between the two peaks in our experiment, comparable to the observed angular resolution, and at higher pressures *β* tends towards 90°, decreasing the separation. We therefore assume a purely orthorhombic structure for this analysis. In both this work, and the static compression experiments of Fontana, other allowed peaks of the high pressure *A*2*/m* structure - (101), (210) and (020) - are not observed, the reason for which is unknown.

From the diffraction peak positions, we can calculate the lattice parameters and therefore the unit cell volumes at each condition reached. The cell volumes are fitted with a Rose-Vinet EOS^33,34^ of the form:

1 $$P({T}_{0},V)=3{B}_{0}[(1-f)/{f}^{2}]\times \exp [1.5({C}_{0}-1)(1-f)]$$

2 $$P(T,V)=P({T}_{0},V)+{\alpha }_{0}{B}_{0}(T-{T}_{0})$$

where *f* = (*V*/*V*_0_)^1/3^. *B*_0_ is the isothermal bulk modulus, which is constrained to literature values^28–30^. *C*_0_ describes the change in bulk modulus with pressure i.e. ${C}_{0}={(\frac{\partial B}{\partial P})}_{0}$, and *α*_0_ is the volumetric thermal expansion coefficient. The fitting parameters are estimated from a least-squares fit, with one-sigma errors quoted.

**Figure 5:**
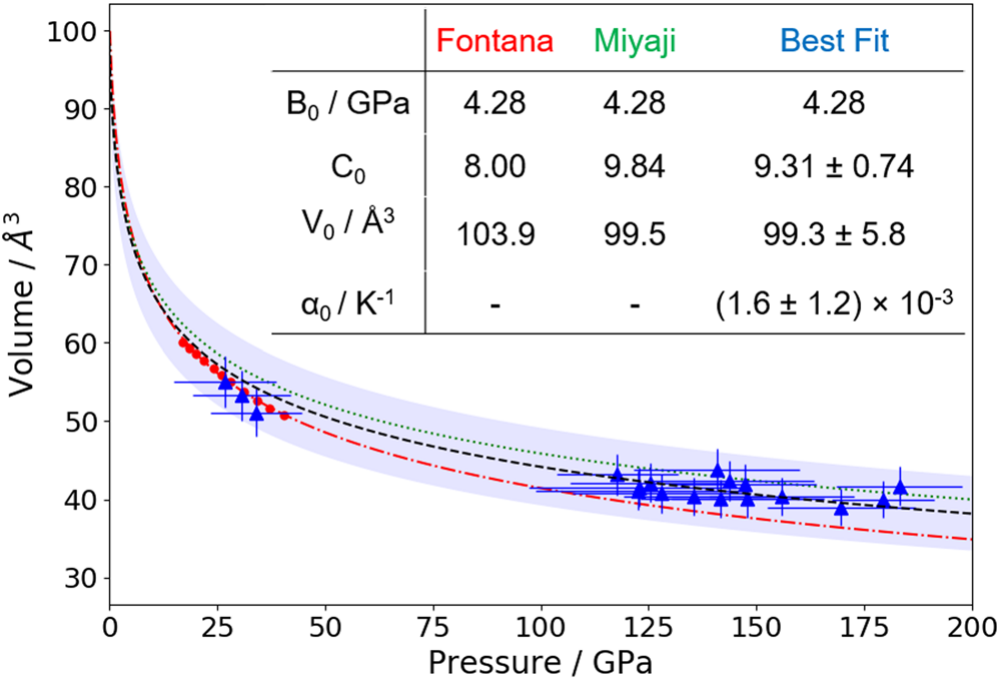
Pressure-volume relations for the CH_2_ monoclinic structure. Red points and dotted line show data and fit from Fontana^30^; green dotted line shows values from Miyaji^29^; blue triangles show data from this work, with effect of temperature subtracted from the pressure; black dashed line shows best fitting EOS, and shaded region the uncertainty. The inset table shows the values used for generating the three lines with the Rose-Vinet EOS and, for our fit, associated uncertainties.

Unlike experiments using static compression, the effect of temperature is significant here, giving an increase in pressure of up to *α*_0_*B*_0_(*T* − *T*_0_) = 10 ± 8 GPa. This was included in the fitting to the Rose-Vinet EOS, but has been subtracted on a shot-by-shot basis for plotting the pressures in Fig. 5, using the temperatures estimated from simulation. The figure therefore shows the cell volumes as a function of the pressures expected at ambient temperature, as this allows direct comparison with prior work. A decrease in the assumed temperature, such as might be indicated by the Hugoniot of Mattsson in Fig. 4, would have the effect of increasing the calculated pressures at *T*_0_, and therefore slightly increasing both *C*_0_ and *V*_0_, although not outside the quoted uncertainties.

It is clear from Fig. 5 that the variation in our dynamical compression data is significantly larger than that from previous work using static compression; the larger uncertainties can be seen in the pressure and particularly the temperature conditions, as well as in the EOS fit. Our results are slightly better fitted by the parameters of Miyaji^29^, who took data at pressures of up to 1 GPa, but extrapolating both fits to the pressures considered, which are much higher than in the original experiments, gives similarly good agreement. The model used for the effect of the temperature, taken from Vinet *et al*.^34^, is a relatively simple one, but the residual of the fit is weakly correlated with temperature. A more complex model is thus not expected to improve the agreement. The deviations may rather reflect the uncertainty in the conditions reached by the laser shock compression, as neither the pressure nor temperature is extracted directly, but estimated from simulations.

For the *A*2*/m* lattice to be observed, there must still be significant numbers of covalently bonded polymer chains. Although the stability of chemical bonding at these conditions seems surprising, structural predictions have previously suggested that molecular and polymeric structures may have favourably low enthalpies, even above 200 GPa^9^. We see in Fig. 4 that the conditions at which this structure occurs are also close to those of a recent model for planetary interiors^35^. Our results therefore imply that, deep in the interior of ‘ice giant’ planets there exists not just carbon-carbon bonding, which has previously been inferred^20,36^, but also carbon-hydrogen bonding. Such chemical processes would have a huge impact on the evolution and behaviour of the mantles of these bodies, since most models assume free hydrogen, in either a metallic^10^ or superionic^7,11^ state. The strong temperature dependence of the chemical structures highlights the importance of better constraining the temperature present inside the planets.

## Summary

In conclusion, we have observed structural order remaining in shock-compressed polyethylene samples at pressures above 200 GPa, verifying *ab initio* predictions. The lattice structure is consistent with the *A*2*/m* phase, which had previously been observed at pressures up to only 40 GPa under static compression. The lattice parameters extracted from the data agree with extrapolations from the lower pressure data, increasing confidence in our identification of the structure. Precise characterization of the melt line was not possible, but for pressures of ~170 GPa, our results suggest that it occurs near 3000 K and increases with pressure, as expected. The conditions at which the structure is observed are potentially relevant to planetary interiors, highlighting the importance of covalent bonding even at high pressure and temperature conditions.
